# An investigation of the process of change in psychopathology and exercise during inpatient treatment for adults with longstanding eating disorders

**DOI:** 10.1186/s40337-018-0201-7

**Published:** 2018-07-01

**Authors:** S. Bratland-Sanda, K. A. Vrabel

**Affiliations:** 1 Department of Sports, Physical Education and Outdoor Studies, University of Southeast Norway, Bø in Telemark, Norway; 2 Research Institute and Department of Eating Disorders, Modum Bad Psychiatric Center, Vikersund, Norway

**Keywords:** Eating disorder, Inpatient, Excessive exercise, Physical activity, Psychiatry

## Abstract

**Background:**

Excessive exercise is recognized as a predictor of poor outcome in eating disorders. However, little is known about how excessive exercise might affect the treatment process. The aim of this study was to describe process of weekly changes in eating disorder psychopathology, general psychopathology and exercise, and the possible interactive effects of excessive exercise on these changes during inpatient treatment of longstanding eating disorders.

**Methods:**

Eighty-four patients meeting the DSM-IV criteria for Anorexia Nervosa, Bulimia Nervosa, or Eating Disorders Not Otherwise Specified received inpatient cognitive-behavioural therapy including, physical activity and nutritional counselling treatment over 12 weeks. Excessive exercise was defined as having ≥6 episodes of driven exercise during week 1 of treatment. Excessive exercisers received one additional session of individual counseling with the clinical exercise physiologist. The study used repeated measurements during treatment and collected measures of eating disorders: psychopathology (EDE-Q), general psychopathology (SCL-5), and frequencies of exercise and body mass index (BMI). Statistical analysis was performed using repeated measures ANOVA.

**Results:**

Both eating disorders and general psychopathology were reduced from admission to discharge in excessive exercisers and non-exercisers. There was an overall interaction effect between time (week) and excessive exercise for the process of exercise and eating disorders psychopathology reduction. This interaction effect was also found in week 10 vs 11 regarding general psychopathology. The excessive exercisers showed steep reduction at first, followed by a smaller increase towards the end of treatment in both eating disorder and general psychopathology; this pattern was not found among the non-exercisers.

**Conclusion:**

The process of change in exercise and psychopathology during inpatient treatment of longstanding eating disorders differs across excessive and non-excessive exercisers. Although excessive exercisers were given special attention for their exercise cognition and behavior during treatment, it is apparent that this part of treatment must be further developed.

## Plain english summary

An excessive amount of exercise is common in eating disorders. It has been shown that excessive exercisers have poorer treatment outcome compared to non-exercisers. Yet, little is known about how excessive exercise might affect process of change that occur during treatment of eating disorders. In this study, level of eating disorders, anxiety and depression, exercise, purging, and use of diuretics/laxatives were obtained weekly in 84 adults admitted to 12-weeks of inpatient eating disorders treatment. The process of change in eating disorders and anxiety/depression during treatment was somewhat different between the excessive exercisers and the non-exercisers. Based on this finding, we need to develop strategies that specifically target excessive exercise during treatment of eating disorders.

## Background

A significant portion of patients admitted to treatment for eating disorders (ED) do not recover despite use of well-established and evidence-based treatment forms such as individual psychotherapy, group-based therapy, and family-based therapy [4, 19, 42]. Attempts have been made to explore factors that predict ED treatment outcome. Studies have suggested that motivation to change and rapid response to treatment predict a good outcome [25, 40], whereas comorbidity (i.e. anxiety and depression), greater ED psychopathology such as weight concern, shape concern, and bulimic episodes, predict a poor outcome [4, 40]. Other studies have also identified excessive levels of exercise as a symptom that occurs alongside more severe ED psychopathology and general psychopathology, predicts a poor outcome of treatment, as well as an increased risk of relapse [8, 22, 32, 36].

In existing literature, several terms and definitions such as excessive exercise, high-level exercise, compulsive exercise, and exercise dependence have been used to explain and define pathological exercise in ED [2]. A more succinct differentiation of these terms have been formed: excessive exercise as the quantitative dimension (i.e. excessive in frequency, duration and/or intensity), as well a compulsive exercise as the qualitative dimension (i.e. motives and attitudes) [2]. A recent study by Young et al. [43] showed that assessments of frequencies of driven exercise such as the exercise questions in the Eating Disorders Examination interview (EDE) and questionnaire (EDE-Q) correlated well with assessments of compulsive exercise. However, when examining exercise during treatment, it is of interest to divide between the cognitions and the actual behaviour. We therefore need to expand knowledge regarding quantity of exercise during inpatient treatment of eating disorders, and how this changes in patients who perform excessive amounts of exercise.

Excessive and/or compulsive exercise has been identified in up to 80% of persons with ED, depending on type of ED diagnosis and duration of the disorder [13, 27, 32]. During the past decade, there has been an increased understanding of the complexity of the function of exercise as a symptom of ED. From the belief that exercise was solely a weight compensatory behavior [11], exercise is now acknowledged as important for suppressing negative affect [7, 8, 23, 24]. In a maintenance model for compulsive exercise, Meyer et al. [27] suggested that there is a reciprocally reinforcing relationship between compulsive exercise and affect regulation. That is, the exercise maintains the function of controlling negative affect, whereas high levels of negative affect seem to increase levels of compulsive and excessive exercise [27]. Likewise, the excessive amounts of exercise can have a negative impact on mood, as seen among e.g. overtrained athletes [1]. A qualitative study among women with Anorexia Nervosa (AN) also showed that the exercise functioned as a distraction and escape, a strategy for reducing embodied distress, a time-out from conflicting thoughts and feelings, and a relation to their sense of self and identity [24]. Dealing with these issues and complexity in treatment seem to improve the success of gaining weight and reduction of compulsive exercise among excessive exercisers [9]. Despite the increased understanding of exercise in ED, Touyz et al. [39] argues that there still is a need for more research in this area. Although Touyz et al. [39] addresses this gap in knowledge primarily in persons with AN, the phenomenon of excessive and/or compulsive exercise is also present in persons with Bulimia Nervosa (BN) and Eating Disorders Not Otherwise Specified (EDNOS). There is therefore a need for such attention across the whole spectrum of ED diagnoses.

In previous studies, changes in ED psychopathology, exercise, and other compensatory behavior during treatment are often assessed at only two times, i.e. at admission and discharge. In one previous study, we assessed physical activity objectively three times during treatment, and found different trends in the weekly physical activity between excessive exercisers and non-exercisers [8]. All patients experienced reduced ED psychopathology from admission to discharge, but the excessive exercisers had higher scores on Eating Disorders Examination interview at both assessment times compared to the non-exercisers [8]. Unfortunately, changes in ED psychopathology were only assessed at admission and discharge in this study. Therefore, it is unknown how change evolved during the treatment period, and if it differed across excessive exercisers and the non-exercisers. This prevents conclusions about how changes in exercise and psychopathology develop over the course of therapy. To build knowledge in this sense we need to monitor the patients thoroughly throughout the entire treatment period, from admission to discharge.

To our knowledge, no studies describe weekly changes in ED psychopathology, general psychopathology, exercise, and other weight compensatory behavior such as purging and use of diuretics/laxatives during the course of ED treatment. Reporting week-to-week changes provides a greater understanding of when change is taking place and the process of this change. Following the logic of evidence-based medicine, the choice of methods and research designs must apply to the current level of knowledge. We therefore justify that there is a need for obtaining descriptive data on the process of change during ED treatment and possible interactions of excessive exercise in this process. Enhancing level of knowledge in this area is important for ED treatment in general, as well as for the treatment of excessive exercisers in particular. Understanding this can directly influence the quality of care delivered to these patients. Such descriptive data can build a foundation and rationale for future experimental studies. The aim of this study is therefore to describe the process of changes in ED psychopathology, general psychopathology, and frequencies of exercise during inpatient treatment of longstanding ED. In view of the paucity of existing evidence, no specific hypotheses were formulated in this regard. Our research questions are as follows: 1) How does ED psychopathology, general psychopathology, and frequency of exercise change by week? and 2) Is there an interaction effect of week and excessive exercise in the process of change in ED psychopathology, general psychopathology, and exercise?

## Methods

The patients in the sample received inpatient treatment within a specialized ED unit at a psychiatric facility. The unit has a nationwide catchment area. All patients have tried therapy locally without satisfactory treatment benefits prior to admission to the unit. A team of independent psychologists and psychiatrists with extensive training and experience in diagnostic assessment evaluated potential participants by conducting a detailed clinical interview and using standardized assessment tools. Using self-assessment instruments, patients reported on general outcome measures at start and end of therapy in addition to outcome on a weekly basis while undergoing inpatient treatment, hence 12 consecutive treatment weeks. This study is approved by the Regional Committee for Medical Ethics in Southern Norway, approval no. 2012/1186b.

### Treatment

Manual, individualized cognitive behavioral therapy (CBT) was developed by the unit, based on CBT for ED. The treatment was an adaptation of outpatient CBT for ED developed by Waller et al. [41]. The treatment was primarily concerned with the processes hypothesized to maintain the patients’ ED psychopathology, aiming to view cognitive processes as central, altering abnormal attitudes about body shape and weight, replacing dysfunctional dieting with normal eating habits, and developing coping skills for resisting bingeing and purging. The key strategy of the treatment was to create a “formula” (or a set of hypotheses) of the maintaining mechanisms of the patients’ psychopathology. The formula was used to identify the features to address in the treatment. An initial personal formula was developed collaboratively with patients at the start of the treatment, and this was revised during the course of the treatment. The aim was to create a tailor-made treatment that fit the individual patient. In addition to individual therapy, patients attended daily group sessions conducted by a multidisciplinary team all trained in CBT, two of which had several years of experience delivering CBT for ED. According to the CBT-principal, patients received psychoeducation, individual goal setting and evaluation, in-vivo exposure to meals, and two weekly group sessions of physical exercise. The therapy lasted for 12 weeks.

The patients who were categorized as excessive exercisers received one individual counseling session with a clinical exercise physiologist. This session lasted approximately 45 min. During this session, an individual plan for reducing excessive exercise was made. The plan was made in agreement with the patient and the therapists, and it was intended for the patient to take ownership over this plan. This session took place during week two in the treatment period.

### Instruments

Eating Disorder Examination Questionnaire version 6.0 (EDE-Q). The EDE-Q (Christopher G. [15]) was used to assess ED psychopathology and generate ED diagnoses. The EDE-Q consists of four subscales: restraint, shape concern, weight concern, and eating concern. A mean value is calculated on a 0–6 point scale. For weekly assessment, the patients were asked about the last 7 days instead of last 28 days. Adequate reliability of the 7-day version has been demonstrated by Rose et al. [31]. EDE-Q has shown adequate psychometric properties across a range of studies [3]. Excessive exercisers were defined as persons with six or more episodes of driven exercise within the past 7 days [6, 12] at baseline, identified by question 18 in the EDE-Q. Frequencies of exercise, purging and use of diuretics/laxatives were identified by questions 16–18 in the EDE-Q.

Symptom Checklist-5 (SCL-5). The SCL-5 is an indicator of global mental distress that has been used as a screening measure of psychological distress in several studies [14, 21, 35, 38]. The SCL-5 has five items: (1) Feeling fearful, (2) Nervousness or shakiness inside, (3) Feeling hopeless about the future, (4) Feeling blue, and (5) Worrying too much about things. Each of the five items is scored on a scale of 1 to 4. The checklist mainly screens for symptoms of anxiety and depression [37]. SCL-5 correlates strongly with SCL-25 (Person’s correlation = 0.92). The recommended cut-off on the SCL-5 total mean score, indicating distress at case-level, is 2.00.

Body Mass Index (BMI). BMI (kg/m^2^) was calculated using height and weekly measured fasting body weight. Patients with a BMI below 20 at admission achieved weight-gain up to the minimum BMI of 20 as recommended by existing treatment guidelines [26].

### Statistical analysis

For the statistical analysis, the IBM SPSS 24.0 was used. Data are presented in mean (SD), and in frequencies and percentage. Independent t-test and chi-squared test were used to examine differences at admission between excessive exercisers and non-exercisers. Missing values in SCL-5, EDE-Q, exercise, purge and use of diuretics/laxatives were replaced with series mean [17]. GLM repeated measure ANOVA is the preferred analysis for variables obtained at more than two assessment times [33]. Factor was time (weeks in treatment), and measures were SCL-5 score, EDE-Q score, weekly episodes of exercise, and BMI. Data on exercise, purging and use of diuretics/laxatives were non-parametric. This data was therefore log transformed for the GLM repeated measurement ANOVA. Due to loss of data with values of zero in log transformation, there was too much data lost regarding purging and use of diuretics/laxatives to continue with the analysis of these variables. They were therefore excluded from the GLM repeated measurement ANOVA, and were instead analysed at admission and discharge with Mann-Whitney U test for two samples (excessive exercisers and non-exercisers). Exercise category (excessive exerciser = yes or no) was selected as between-subject factor. For changes in BMI, BMI < 20 (yes or no) was selected as a between-subject factor. Based on recommendations by Girden [18], we used the Greenhouse-Geisser correction for Mauchly’s test of sphericity. Bonferroni post hoc test was obtained to depict where the differences occurred. We reported Type III sum of squares, degrees of freedom (df), *F*-value, significance level, and effect size (Partial Eta Squared). The Partial Eta Squared effect size was classified as small (.01), medium (.06) and large (.14) based on recommendations from Miles et al. [28]. Significance level was .05.

## Results

### Descriptive data

A total of 23% of the 84 patients were defined as excessive exercisers. Excessive exercising was more common among patients diagnosed with AN compared to BN and EDNOS, and less common among patients diagnosed with BN compared to AN and EDNOS (Table 1). Thirty-three percent of the patients had a BMI below 20 at baseline and hence had weight gain as one of their treatment goals (Table 1).

**Table 1:** Descriptive data

	Excessive exercisers (*n* = 19)	Non-exercisers (*n* = 65)	Difference EE/non-E	Total (*n* = 84)
	*Mean (SD)*	*Mean (SD)*	*t-value*	*Mean (SD)*
Age (yrs)	27.7 (8.0)	28.2 (7.7)	0.21	28.1 (7.7)
BMI (kg/m^2^)	20.8 (3.6)	21.8 (5.2)	0.82	22.5 (4.8)
ED duration (yrs)	10.6 (7.7)	12.9 (8.4)	1.04	12.4 (8.3)
Inpatient stays (number)	1.0 (1.2)	1.3 (2.0)	0.60	1.2 (1.8)
SCL-5	2.7 (0.7)	2.4 (0.9)	1.62	2.5 (1.0)
EDE-Q	3.9 (1.1)	3.7 (1.1)	0.81	3.7 (1.1)
Exercise (episodes/week)	7.2 (2.4)	1.1 (1.5)	10.38***	2.5 (0.9)
Purging (episodes/week)	5.5 (10.9)	6.0 (9.6)	0.17	5.9 (9.9)
Diuretics/laxatives (episodes/week)	0.4 (1.6)	0.3 (0.9)	0.25	0.3 (1.1)
	n (%)	n (%)	χ^2^	n (%)
Diagnosis: AN	9 (43)	12 (57)	7.51**	21 (25)
Diagnosis: BN	5 (12)	38 (88)	6.33*	43 (51)
Diagnosis: EDNOS	5 (25)	15 (75)	0.34	20 (24)
BMI < 20 kg/m^2^	7 (37)	21 (32)	0.14	28 (33)
Self-mutilation	6 (32)	31 (48)	1.55	37 (44)

*EE* excessive exercisers, *Non-E* non-exercisers, *BMI* body mass index, *ED* eating disorders, *SCL-5* Symptom Check List – 5 items, *EDE-Q* Eating Disorders Examination – Questionnaire, *AN* Anorexia Nervosa, *BN* Bulimia Nervosa, *EDNOS* Eating Disorders Not Otherwise Specified

*p < .05. **p < .01. ***p < .001

### Week-to-week changes during treatment

**Fig. 1:**
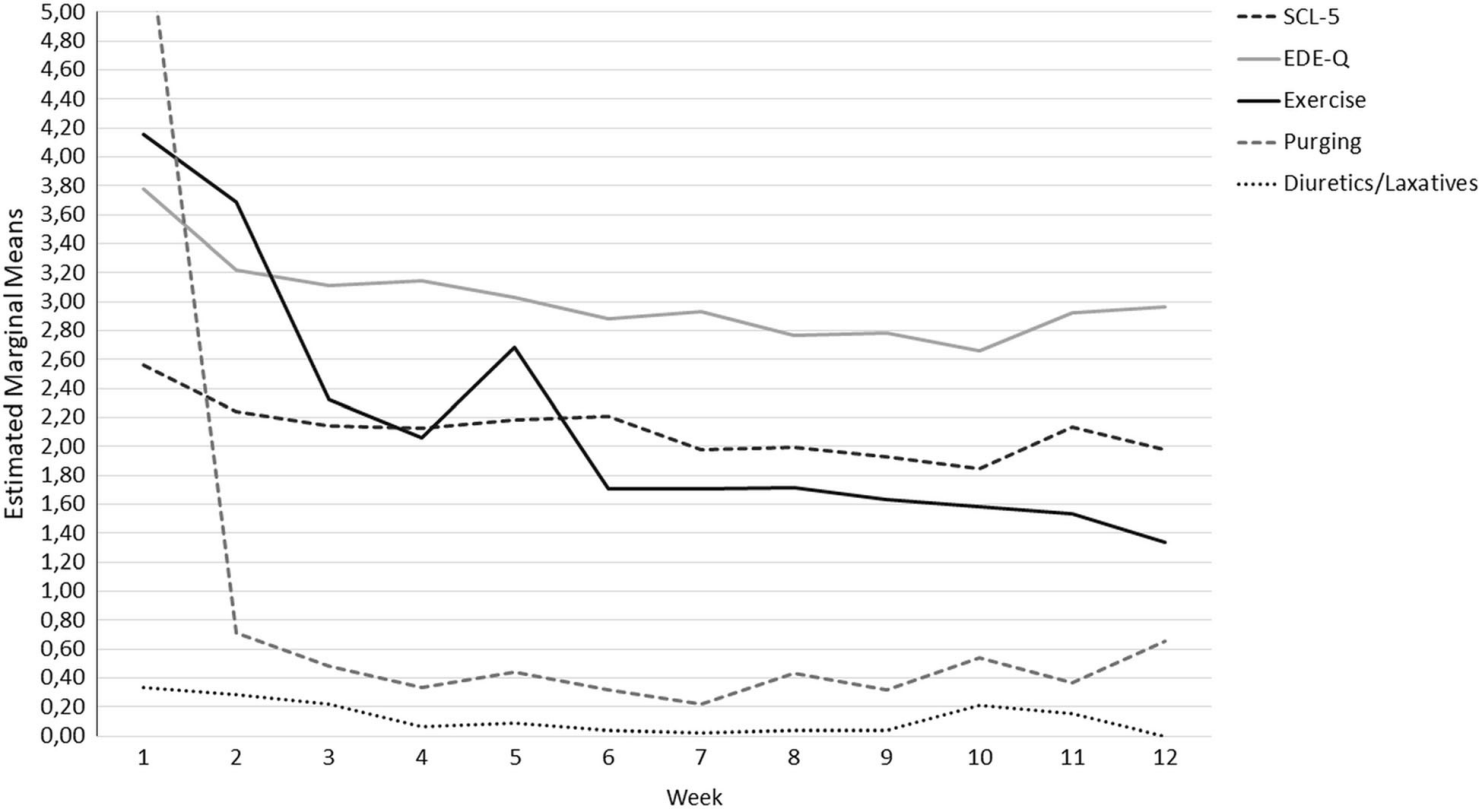
Process of change in SCL-5 (score range 0–4), EDE-Q (score range 0–5), and episodes per week of exercise, purging and use of diuretics/laxatives

During treatment, there was a reduction in EDE-Q, SCL-5, and exercise, whereas there was an overall increase in BMI (Table 2). These changes were significant for both excessive exercisers and non-exercisers. The lowest scores in SCL-5 and EDE-Q were observed in week 10 (Fig. 1). From week 10 vs. 11, there was a significant increase in SCL-5 (*F* = 6.06, *p* = .02) and in EDE-Q (*F* = 5.72, *p* = .02). The profile of change in exercise showed significant reduction in weekly exercise from week 2 vs. 3 (*F* = 9.65, *p* = .003) and an increase in weekly exercise from week 5 vs. 6 (*F* = 4.09, *p* = .046, Fig. 1). The BMI among the patients with a BMI < 20 at admission increased by 20% during treatment (from 17.13–20.49 kg/m^2^, *F* = 5.75, *p* = .001, *ES* = .06). Weekly information on episodes of purging and use of diuretics/laxatives is shown in Fig. 1; the Mann Whitney U test for two samples showed no differences in these variables between the excessive exercisers and the non-exercisers during the treatment period.

**Table 2:** Changes during inpatient treatment of longstanding eating disorders. A) Effect of time, B) interaction effect of time and excessive exercise

	Type III of sum squares	df	Mean squares	*F*-value	*p*-value	Partial eta squared
*A. Week*						
SCL-5	22.83	6.7	3.40	3.97	<.001***	.05
EDE-Q	54.58	5.94	9.18	10.56	<.001***	.11
Exercise (episodes/week)^a^	1.68	6.95	0.24	3.61	.002**	.21
BMI (kg/m^2^)	160.61	2.54	63.37	3.84	.02*	.05
*B) Week* ^*^ *Excessive_exercise*						
SCL-5	8.45	6.73	1.25	1.47	.18	.02
EDE-Q	12.43	5.94	2.09	2.40	.03*	.03
Exercise (episodes/week)^a^	1.11	6.95	0.16	2.39	.027	.15
BMI (kg/m^2^)	43.95	2.54	17.34	1.05	.364	.01

*SCL-5* Symptom Check List – 5 item, *EDE-Q* Eating Disorders Examination Questionnaire, *Df* degree of freedom

^a^log transformed data

*p < .05. **p < .01. ***p < .001

**Fig. 2:**
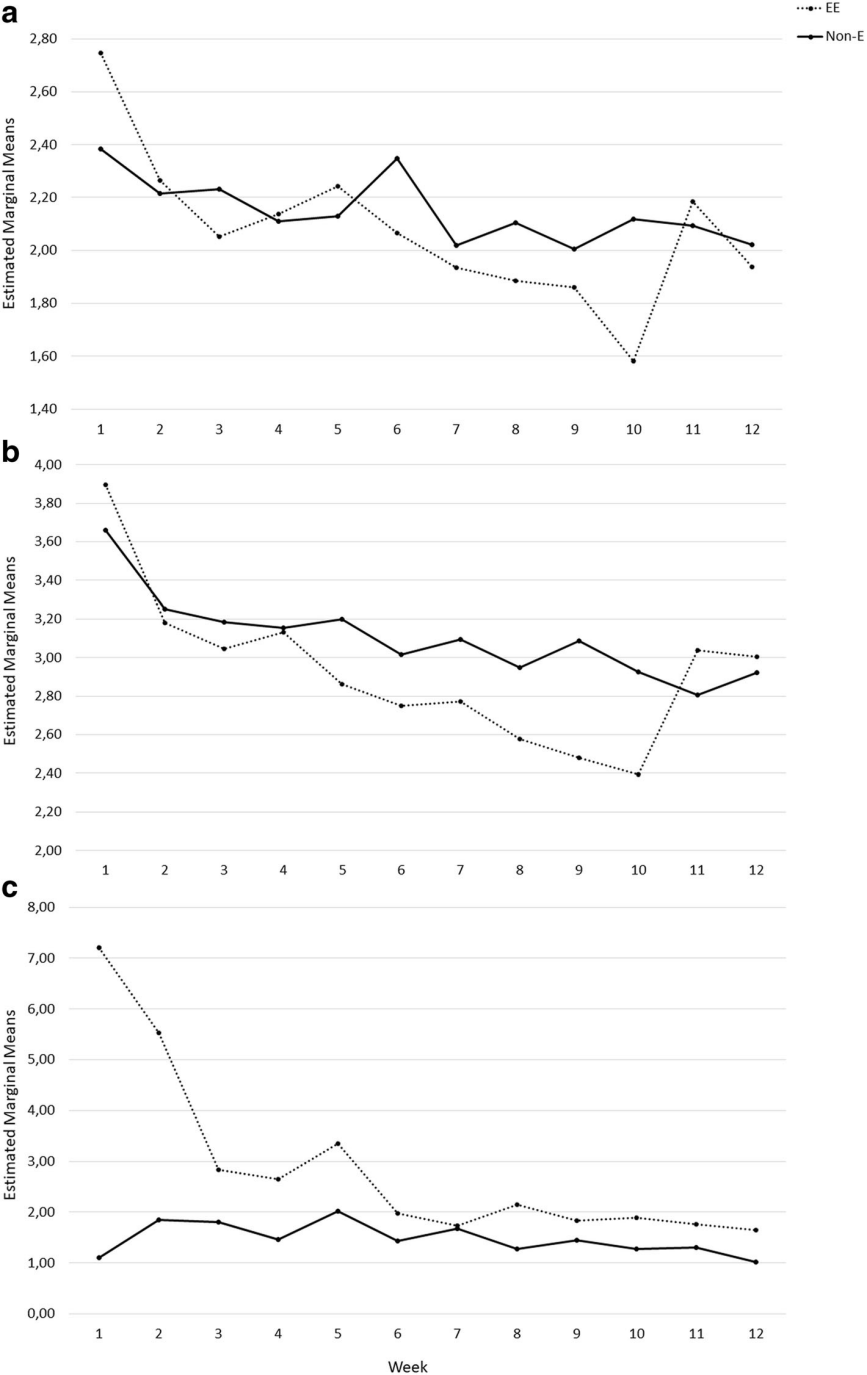
Interaction effect of time (week) and exercise category (excessive exercise:EE, non-exercise: non-E) on the process of change in SCL-5 (**a**), EDE-Q (**b**) and exercise (**c**) during inpatient treatment of longstanding eating disorders

No interaction effects found between week number and excessive exercise were detected for BMI, but there was an interaction effect between week number and BMI < 20 in the process of BMI change during treatment (*F* = 5.75, *p* = .001, *ES* = .07). There was an overall interaction effect between week number and excessive exercise for EDE-Q and weekly episodes of exercise (Table 2). Although there was not an overall interaction effect between week number and excessive exercise for changes in SCL-5 score, post-hoc tests showed that there was a borderline significant interaction effect in week 9 vs. 10 (*F* = 3.80, *p* = .055, *ES* = .04), and significant interaction effect in week 10 vs. 11 (*F* = 7.26, *p* = .009, *ES* = .08) (Fig. 2a). The same interaction effect pattern in week 10 vs. 11 was found for EDE-Q (*F* = 12.01, *p* < .001, *ES* = .13) (Fig. 2b). The interaction effect of week number and excessive exercise on weekly exercise was found significant in week 1 vs. 2 (*F* = 5.85, *p* = .018, *ES* = .07), 2 vs. 3 (*F* = 9.00, *p* = .004, *ES* = .10), and borderline significant in week 7 vs. 8 (*F* = 3.85, *p* = .053, *ES* = .05) (Fig. 2c).

## Discussion

The main finding of this observational study was the overall interaction effect between week number and excessive exercise for changes in EDE-Q during inpatient treatment. Furthermore, there was also an interaction effect between week number and excessive exercise for both EDE-Q and SCL-5 in week 10 vs. 11, which is the second last week before discharge. As Fig. 2a and b shows, the excessive exercisers showed an increase in both EDE-Q and SCL-5 from week 10 to 11. This pattern was not observed among the non-exercisers. Although differences between excessive exercisers and non-exercisers were in accordance with findings from previous studies [8, 32, 34], this is the first study that reports the process of change in detail to our knowledge. Since change in BMI was similar between the excessive exercisers and the non-exercisers, the most plausible explanation for the pattern observed among the excessive exercisers is the use of exercise for regulation of negative affect. Due to the suppressing function of exercise on e.g. anxiety, exercise withdrawal can possibly result in increased levels of anxiety [27]. It could be that the excessive exercisers have successfully postponed or abstained from exercise in the major part of the inpatient stay. However, the experience of negative emotion inevitably increased at the end of the treatment. In addition, the end of an inpatient stay is always challenging for patients since they are about to discharge from a treatment context in which they have made important changes. The uncertainty of a new life after discharge can increase both ED and general psychopathology for patients, especially if the treatment context is not especially focused on addressing this challenge. It could be that excessive exercise is easily overlooked in a treatment context since this is often considered as compensatory behaviour, and that the complexity of the exercise with regards to compulsivity is ignored. It is easier to focus on e.g. purging and the misuse of laxatives as compensating behaviour since this is typically considered as abnormal behaviour. Management of exercise in patients may have been ‘bundled’ together with attempts to treat other forms of purging and compensation (C. G. [16]). Studies have shown that the classification of exercise as a purely compensatory role is inadequate, as the nature of both excessive and compulsive exercise is more complex. There is reliable evidence that the amount and intensity of exercise undertaken is not significantly associated with drive for thinness, which would be the case if exercise were solely a compensatory behaviour [2, 5, 7, 29].

The changes in ED psychopathology is comparable to previous studies [8, 30]. It is interesting that the patients with BMI below 20 managed to increase BMI to the extent that was observed. Previous studies show that weight gain above 17.5 during treatment is difficult [20],

### Strengths and limitations of the study

ED psychopathology, general psychopathology, and episodes of exercise were assessed weekly, and adequate methods were utilized to monitor the process thoroughly. Findings from such studies directly inform therapists on variables relevant at the level where clinical decisions are met. However, we have to bear in mind that this is purely a descriptive, observational study. We therefore cannot make assumptions about causality. The process was studied on a weekly basis, and more or less frequent assessments could be associated with different results. In addition, the measurement of exercise is purely self-fulfilling and not objectively measured. This could affect the result in a way that the patients under-report, e.g. frequency of excessive exercise [7]. The EDE-Q exercise question used to assess excessive exercise correlates well with measures of compulsive exercise. However, it does not capture the complexity of exercise cognitions. Future process studies need to examine both the excessive and compulsive nature of exercise in ED. The patients studied exhibited high symptom severity; they are a selected group, given the admission criteria for treatment at this specialized unit. Such severity and selection might affect the external validity of the findings. However, it might be that the level of symptomatology amplifies core traits present in ED, making the sample especially interesting to study. Nevertheless, the sample is representative of a group of patients that frequently do not benefit from treatment. This is evident by how frequently patients reported to have been in therapy prior to inclusion in the current treatment. Furthermore, all diagnostic categories were represented, reflecting the diagnostic distribution commonly seen in clinical practice.

Previous studies have shown that excessive exercisers across diagnoses require a longer length of hospitalisation than non-exercisers [34]. In addition, excessive exercise is predictive of earlier relapse time and poor long-term outcome at follow-up [10, 36]. Our study can explain some of these previous findings. Patients with excessive exercise as a problem are discharged the moment they are highly activated by both ED and general psychopathology due to the fact that they are obviously vulnerable for relapse and poorer long-term follow-up. The difference we display between excessive exercisers and non-exercisers in this study can be an argument for a varied duration of inpatient stay.

### Implications

As elaborated above, our study invites an increased focus on excessive exercise during inpatient treatment. We argue that special attention to exercise towards the end of an inpatient stay seems important, as it seems that both ED and general psychopathology increase in the end for patients practicing excessive exercise. Experimental studies that integrate such attention with existing therapy must be developed; these studies should include a follow-up after treatment discharge. Moreover, future studies should investigate the interaction between excessive exercise and treatment outcome across therapies, therapy contexts, as well as type and severity of disorders.

## Conclusion

The process of change in exercise and psychopathology during inpatient treatment of longstanding eating disorders interacts with time and excessive exercising. Although excessive exercisers were given special attention for their exercise cognition and behaviour during treatment, it seems that this part of treatment must be further developed.

## Abbreviations

AN: Anorexia nervosa
BMI: Body mass index
BN: Bulimia Nervosa
DSM-IV: Diagnostic and statistical manual 4th Ed.
ED: Eating disorder
EDE-q: Eating disorder examination questionnaire
EDNOS: Eating disorder not otherwise specified
SCL-5: Symptom Checklist-5

## References

[1] Adams J, Kirkby R (2001). Exercise dependence and overtraining: the physiological and psychological consequences of excessive exercise. Sports Med Train Rehab.

[2] Adkins EC, Keel PK (2005). Does “excessive” or “compulsive” best describe exercise as a symptom of bulimia nervosa?. Int J Eat Disord.

[3] Berg KC, Peterson CB, Frazier P, Crow SJ (2012). Psychometric evaluation of the eating disorder examination and eating disorder examination-questionnaire: a systematic review of the literature. Int J Eat Disord.

[4] Berkman ND, Lohr KN, Bulik CM (2007). Outcomes of eating disorders: a systematic review of the literature. Int J Eat Disord.

[5] Boyd C, Abraham S, Luscombe G (2007). Exercise behaviours and feelings in eating disorder and non-eating disorder groups. Eur Eat Disord Rev.

[6] Bratland-Sanda S, Martinsen EW, Rosenvinge JH, Ro O, Hoffart A, Sundgot-Borgen J (2011). Exercise dependence score in patients with longstanding eating disorders and controls: the importance of affect regulation and physical activity intensity. Eur Eat Disord Rev.

[7] Bratland-Sanda S, Sundgot-Borgen J, Ro O, Rosenvinge JH, Hoffart A, Martinsen EW (2010). “I'm not physically active - I only go for walks”: physical activity in patients with longstanding eating disorders. Int J Eat Disord.

[8] Bratland-Sanda S, Sundgot-Borgen J, Ro O, Rosenvinge JH, Hoffart A, Martinsen EW (2010). Physical activity and exercise dependence during inpatient treatment of longstanding eating disorders: an exploratory study of excessive and non-excessive exercisers. Int J Eat Disord.

[9] Calogero RM, Pedrotty KN (2004). The practice and process of healthy exercise: an investigation of the treatment of exercise abuse in women with eating disorders. Eat Disord.

[10] Carter JC, Blackmore E, Sutandar-Pinnock K, Woodside DB (2004). Relapse in anorexia nervosa: a survival analysis. Psychol Med.

[11] DalleGrave R, Calugi S, Marchesini G (2008). Compulsive exercise to control shape or weight in eating disorders: prevalence, associated features, and treatment outcome. Compr Psychiatry.

[12] Davis C, Katzman D. K, Kaptein S, Kirsh C, Brewer H, Kalmbach K, Kaplan AS. The prevalence of high-level exercise in the eating disorders: etiological implications. Compr Psychiatry*,* 1997;38(6), 321–326. 10.1016/s0010-440x(97)90927-5 9406737

[13] Davis C, Kennedy SH, Ravelski E, Dionne M (1994). The role of physical activity in the development and maintenance of eating disorders. Psychol Med.

[14] Eriksen W, Tambs K, Knardahl S (2006). Work factors and psychological distress in nurses’ aides: a prospective cohort study. BMC Public Health.

[15] Fairburn CG (2008). Cognitive behavior therapy and eating disorders.

[16] Fairburn CG, Cooper Z, Shafran R (2003). Cognitive behaviour therapy for eating disorders: a “transdiagnostic” theory and treatment. Behav Res Ther.

[17] Gad AM, Abdelkhalek RHM (2017). Imputation methods for longitudinal data: a comparative study. Int J Stat Distrib Appl.

[18] Girden ER (1992). ANOVA: repeated measures.

[19] Grenon R, Schwartze D, Hammond N, Ivanova I, McQuaid N, Proulx G, Tasca GA (2017). Group psychotherapy for eating disorders: a meta-analysis. Int J Eat Disord.

[20] Halvorsen I, Tollefsen H, Rø Ø (2016). Rates of weight gain during specialised inpatient treatment for anorexia nervosa. Adv Eat Disord.

[21] Holm M, Tyssen R, Stordal KI, Haver B (2010). Self-development groups reduce medical school stress: a controlled intervention study. BMC Med Educ.

[22] Kaye WH, Gwirtsman H, George T, Ebert MH, Petersen R (1986). Caloric consumption and activity levels after weight recovery in anorexia nervosa: a prolonged delay in normalization. Int J Eat Disord.

[23] Keyes A, Woerwag-Mehta S, Bartholdy S, Koskina A, Middleton B, Connan F (2015). Physical activity and the drive to exercise in anorexia nervosa. Int J Eat Disord.

[24] Kolnes L-J, Rodriguez-Morales L (2016). The meaning of compulsive exercise in women with anorexia nervosa: an interpretative phenomenological analysis. Ment Health and Phys Act.

[25] Linardon J, Brennan L, de la Piedad Garcia X (2016). Rapid response to eating disorder treatment: a systematic review and meta-analysis. Int J Eat Disord.

[26] Lund BC, Hernandez ER, Yates WR, Mitchell JR, McKee PA, Johnson CL (2009). Rate of inpatient weight restoration predicts outcome in anorexia nervosa. Int J Eat Disord.

[27] Meyer C, Taranis L, Goodwin H, Haycraft E (2011). Compulsive exercise and eating disorders. Eur Eat Disord Rev.

[28] Miles J, Shevlin M, Sage (2015). Applying regression & correlation : a guide for students and researchers.

[29] Mond JM, Hay PJ, Rodgers B, Owen C (2006). An update on the definition of “excessive exercise” in eating disorders research. Int J Eat Disord.

[30] Ro O, Martinsen EW, Hoffart A, Rosenvinge JH (2004). Short-term follow-up of adults with long standing anorexia nervosa or non-specified eating disorder after inpatient treatment. Eat Weight Disord.

[31] Rose JS, Vaewsorn A, Rosselli-Navarra F, Wilson GT, Weissman RS (2013). Test-retest reliability of the eating disorder examination-questionnaire (EDE-Q) in a college sample. J Eat Disord.

[32] Shroff H, Reba L, Thornton LM, Tozzi F, Klump KL, Berrettini WH (2006). Features associated with excessive exercise in women with eating disorders. Int J Eat Disord.

[33] Singh V, Rana RK, Singhal R (2013). Analysis of repeated measurement data in the clinical trials. J Ayurveda Integr Med.

[34] Solenberger SE (2001). Exercise and eating disorders: a 3-year inpatient hospital record analysis. Eat Behav.

[35] Strand BH, Dalgard OS, Tambs K, Rognerud M (2003). Measuring the mental health status of the Norwegian population: a comparison of the instruments SCL-25, SCL-10, SCL-5 and MHI-5 (SF-36). Nord J Psychiatry.

[36] Strober M, Freeman R, Morrell W (1997). The long-term course of severe anorexia nervosa in adolescents: survival analysis of recovery, relapse, and outcome predictors over 10-15 years in a prospective study. Int J Eat Disord.

[37] Tambs K, Moum T. How well can a few questionnaire items indicate anxiety and depression? Acta Psychiatr Scand. 1993;87 10.1111/j.1600-0447.1993.tb03388.x. 10.1111/j.1600-0447.1993.tb03388.x 8517178

[38] Tambs K, Ronning T, Prescott CA, Kendler KS, Reichborn-Kjennerud T, Torgersen S, Harris JR (2009). The Norwegian Institute of Public Health twin study of mental health: examining recruitment and attrition bias. Twin Res Hum Genet.

[39] Touyz S, Hay P, Noetel M (2017). Is the neglect of exercise in anorexia nervosa research a case of “running out” of ideas or do we need to take a “LEAP” of faith into the future?. J Eat Disord.

[40] Vall E, Wade TD (2015). Predictors of treatment outcome in individuals with eating disorders: a systematic review and meta-analysis. Int J Eat Disord.

[41] Waller G, Cordery H, Corstorphine E, Hinrichsen H, Lawson R, Mountford V, Russell K. Cognitive behavioral therapy for eating disorders: A comprehensive treatment guide: Cambridge University Press. 2007

[42] Wilson GT, Grilo CM, Vitousek KM (2007). Psychological treatment of eating disorders. Am Psychol.

[43] Young S, Touyz S, Meyer C, Arcelus J, Rhodes P, Madden S, Hay P. Validity of exercise measures in adults with anorexia nervosa: the EDE, compulsive exercise test and other self-report scales. Int J Eat Disord*,* 2017;50(5):533–541. 10.1002/eat.22633 10.1002/eat.22633 27696468

